# Evaluation of community knowledge and attitude toward COVID-19: the case of Hawassa city, Sidama, Ethiopia

**DOI:** 10.3389/fpubh.2024.1286181

**Published:** 2024-02-13

**Authors:** Birhanu Betela Warssamo

**Affiliations:** Department of Statistics, College of Natural and Computational Science, Hawassa University, Hawassa, Ethiopia

**Keywords:** knowledge, attitude, COVID-19, community, Hawassa city, Ethiopia

## Abstract

**Background:**

Scientific information on the knowledge and attitude of the community toward the COVID-19 pandemic is a vital step for effective control measures. This study aimed to investigate the level of knowledge and attitude of Hawassa city residents toward COVID-19 and the interaction among knowledge and attitude toward COVID-19.

**Methods:**

A community-based cross-sectional study with stratified random sampling was used from June 2020 to August 2020. Five hundred and eighty-seven residents were selected for the study, which aimed to evaluate their knowledge and attitude toward COVID-19 using a standardized structured questionnaire. Data were collected using face-to-face interviews that strictly follow the WHO and the Ministry of Health Ethiopia guidelines for COVID-19 prevention. Attitude and knowledge were categorized based on the mean score value. Descriptive statistics and two independent multiple logistic regressions were applied to identify the possible determinants of knowledge and attitude using SPSS version 20 set at 95% CIs with a value of p of <0.05 considered statistically significant.

**Results:**

In total, 61.7% of the participants were knowledgeable about the virus and 65.9% had a positive attitude toward COVID-19. Educational level with the categories of second degree and above (AOR = 29.709, 95% CI = 1.239–712.55), first degree (AOR = 3.476, 95% CI = 3.278–22.02), certificate/diploma (AOR = 1.062–18.24, 95% CI = 1.062–18.24), and grade 12 completed (AOR = 1.903, 95% CI = 2.12–6.809); employment status of respondents who were self-employed (AOR = 9.545, 95% CI = 1.165–78.173) and employed respondents (AOR = 10.053, 95% CI = 1.783–56.673); reading interest with categories always (AOR = 34.45, 95% CI = 26.608–4462.226) and sometimes (AOR = 17.24, 95% CI = 17.213–1661.966); and habit of using social media with categories always (AOR = 38.708, 95% CI = 5.086–294.610) and sometimes (AOR = 3.432, 95% CI = 3.504–23.378) were the significant explanatory variables that were correlated with knowledge of the respondents.

**Conclusion:**

Although the knowledge and attitude of respondents toward COVID-19 were positive, there is a need to use more effective strategies to improve their knowledge and attitude toward COVID-19, and increasing knowledge on preventive behaviors among the community was mandatory to attain better results. The educational level, use of social media, and reading habits of the respondents appear to play significant roles in determining their level of knowledge and attitude toward COVID-19.

## Introduction

SARS-CoV-2 is a family of viruses that can cause illnesses such as common cold, camel flu, and SARS. In 2019, a new coronavirus (SARS-CoV-2) was identified as the cause of the COVID-19 disease outbreak that originated in Wuhan, China and expanded across China and other countries. Most people contaminated by the COVID-19 virus experience light*-*to-mild respiratory sickness and get better without needing special treatment. Adults and those with underlying medical problems such as chest pains, diabetes, long-term respiratory disease, and cancer are more likely to experience severe sickness (1).

The virus that causes COVID-19 is part of a huge family of single-stranded ribonucleic acid viruses that cause sickness, starting as a viral rhinitis to SARS (2). Signs and indicators of COVID-19 may come into view 2–14 days after infection. General signs and symptoms can include fever, cough, tiredness, shortness of breath or difficulty breathing, muscle aches, headache, chest pain, loose stools, pink eye (conjunctivitis), runny nose, and sore throat (3). The aged population and patients with coexisting or co-occurring conditions are more susceptible to being infected and are, in addition, more prone to severe problems, which may be related to adult respiratory distress syndrome and hypercytokinemia (4).

Till the time of this study being conducted, from June 2020 to August 2020, there was no confirmed cure or vaccine obtained for COVID-19. Infection control measures such as washing hands with soap, keeping distance, and wearing a mask were the main interventions to reduce the spread of the virus in the community (5). Public knowledge of the virus plays an excellent role in limiting its spread. Vaccine or medicine development is expected to take a number of days or years, and thus, administration of the pandemic depends first and foremost on communities’ adherence to the optional measures in use (6).

Experts believe that the virus that causes COVID-19 spreads mainly from individual to individual via droplets and from an infected person (7). Adults and sick persons with pre-existing diseases (such as congenital heart disease, respiratory tract anomaly, abnormal hemoglobin level, high blood pressure, obesity, heart disease, and lung disease) are identified as possibly having a risk of harsh malady and death (8, 9). Till the time of this study, scientists have not obtained an antitoxin healing drug or immunization for COVID-19 (2). The World Health Organization (WHO) suggests the elimination of person-to-person transference by avoiding close contact with others, wearing a mask, allowing rooms to circulate air, keeping away from traffic, washing hands, and coughing into a bent elbow or tissue (1).

Knowledge about the infection plays a fundamental role in limiting the expansion of disease to the community. Organization of the disaster depends mainly on people’s adherence to the optional measures taken. These measures are largely affected by the knowledge, attitude, and practice (KAP) of the public (6). Measuring communities’ knowledge and attitudes is vital in recognizing gaps and supporting ongoing prevention efforts. To the best of the author’s knowledge, covariates significantly determining the level of knowledge and attitude of the general population of Hawassa city and the interaction among knowledge and attitude toward COVID-19 have not been assessed. Specifically, no study has been conducted on the level of knowledge and attitude toward COVID-19 among the general population of Hawassa city, Sidama, Ethiopia. Thus, this study aimed to investigate the level of knowledge and attitude of the general population of Hawassa city on COVID-19 and the interaction among knowledge and attitude toward COVID-19.

## Methods and materials

### Study design

A community-based cross-sectional study was conducted.

### Data gathering and quality control

The information was gathered using a pretested standardized questionnaire from 3 June 2020 to 30 August 2020. Data were collected using face-to-face interviews that strictly follow the WHO and the Ministry of Health Ethiopia guidelines for COVID-19 prevention. After checking the validity of the questionnaire using 10% of the sample size, which was not included in the analysis later, the survey was settled. The questionnaire was translated into local languages (Sidamic and Amharic), and during data collection, information was checked for completeness each day by supervisors and investigators. Data were used to assess internal consistency and reliability using Cronbach’s alpha.

### Sampling design

The sampling technique used for this study was the stratified sampling method. The strata used were sub-cities of Hawassa city, Sidama region administrations.

### Target population

The target populations were residents of Hawassa city, Sidama, Ethiopia aged 18 years or older who understood the content of the questionnaire and agreed to participate in the study. Participation in the study was on a voluntary basis, and informed written consent was given to the participants.

### Sample size determination

A simple random sampling method was used using the formula (10)


$$n=\frac{\sum_{i=1}^{k}\frac{{N_{i}}^{2}p\left(1-p\right)}{W_{i}}}{\frac{N^{2}d^{2}}{Z_{\frac{\alpha }{2}}}+Np\left(1-p\right)},$$

where n refers to the required total sample size, N refers to the total number of households (targeted residents) in Hawassa city, Z refers to the inverse of the standard normal cumulative distribution that corresponds to the 5% level of confidence (Z = 1.96), k refers to the total number of sub-cities (strata) in Hawassa city administration (*k* = 7), N_i_ refers to the number of households in each sub-city (for i = 1, 2, 3, 4, 5, 6, 7), W_i_ refers to the estimated proportion of N_i_ to N (sub-city households to the total number of households in Hawassa city), *p* refers to the probability of knowledge and attitude on COVID-19, and d refers to the level of precision (sampling error). Using proportional allocation, the subsample size from each sub-city is given below (Table 1).

**Table 1:** Sample size by sub-cities.

i	Sub-cities	Total number of households in each sub-city (*N*_i_)	*W*_i_	*n*_i_
1	Addis Ketema	2,321	0.092	54
2	BahilAdarash	1,387	0.055	32
3	Hayik Dar	1,924	0.076	45
4	MehalKetema	1,726	0.069	40
5	Menaheriya	4,235	0.169	99
6	Misrak	1,492	0.059	34
7	Tabour	12,099	0.480	283
Total		25,184	1	587

The probability of success was 0.5, which was determined via a pilot study. The level of precision preferred for this study was 4%. The desired sample size from the target population was 587.

### Data processing and analysis

Data were entered as input to SPSS version 20 for cleaning and analysis. Data were presented using both descriptive and inferential statistics. Variables with a *p*-value less than 0.05 in the bivariate analysis were included in the multivariate analysis. Multivariate logistic regression analyses were employed at a 95% confidence interval to determine the presence of an association between independent variables with knowledge and attitude. A *p*-value of <0.05 at a 95% CI was taken as statistically significant. The Kaiser–Meyer–Oklkin (KMO) measure was issued to check the validity of the items and should be more than 0.6. Cronbach’s α was determined to check internal consistency.

#### Operational definitions

Good knowledge: Participants who scored the mean value or above for the given knowledge-based questions.

Poor knowledge: Participants who scored below the mean value for the given knowledge-based questions.

Positive attitude: Participants who scored above the mean value for the given attitude-related question.

Negative attitude: Participants who scored below the mean value for the given attitude-related questions.

### Ethics approval and consent to participants

Ethical clearance (DRBH/125/2020) was obtained from the Department Review Board of Hawassa University. The reason and significance of the study were explained, and informed written consent was obtained from the respondents before conducting the study. All of the data collectors strictly followed the WHO and the Ministry of Health Ethiopia guidelines for COVID-19 prevention.

## Results

### Sociodemographic characteristics of the study participants

A total of 587 study participants with a 100% response rate completed the questionnaire. There were 315 (53.7%) male participants. In terms of the education, 12.1% of the study participants were illiterate, and in terms of education level, 11.6% of them had a second degree and above (Table 2).

**Table 2:** Sociodemographic characteristics of the study participants in Hawassa city, Sidama region, Ethiopia, 2021 (*n = 587*).

Variables	Frequency (*N* = 587)	Percentage (%)
**Age in years**		
18–28 28–38 38–48 Above 48	75 285 123 104	12.8 48.6 21 17.7
**Gender**		
Man Woman	315 272	53.7 46.3
**Marital status**		
Single Married Divorced	213 258 116	36.3 44 19.8
**Educational level**		
No formal education Up to and 12 completed Certificate/diploma Degree M.Sc. and above	71 99 158 191 68	12.1 16.9 26.9 32.5 11.6
**Employment status**		
Unemployed Employed Self-employed	154 292 141	26.2 49.7 24.0
**Monthly income in birr**		
Under 1,000 1,000–3,000 3,001–6,000 Above 6,000	116 131 207 133	19.8 22.3 35.3 22.7
**Accommodation**		
Living alone Not living alone	181 406	30.8 69.2
**Total family size** 1–4 5–8 Above 8	182 268 137	31 45.7 23.3
**Reading habit**		
Rarely Sometimes Always	183 167 237	31.2 28.4 40.4
**Use of media**		
Rarely Sometimes Always	103 183 301	17.5 31.2 51.3

### Respondents’ knowledge regarding COVID-19

The average COVID-19 knowledge score was 15.425 (S.D. = 3.12, min = 0, max = 25). Cronbach’s α for the knowledge scale was 0.770, indicating that the questionnaires were reliable. Furthermore, the KMO values were 0.759, which shows that the criteria of validity are met. All the items in knowledge satisfied the standard loading value of >0.40. The average COVID-19 knowledge score was 15.425 (SD = 3.12, min = 0, max = 25). Although all participants in the city, 100%, heard about COVID-19, only approximately 61.7% of the respondents were aware of the disease, and the remaining 38.3% were not aware. Approximately 56.4% of the respondents were aware that the disease is viral, and 59.6% were informed with the intention that children and youngsters should take action to prevent infection by COVID-19. In total, 20.3% of the study participants believe that children are not at greater risk for COVID-19 than adults (Table 3).

**Table 3:** Participants’ knowledge about COVID-19 in Hawassa city, Sidama, Ethiopia, 2020 (*n* = 587).

Items related to knowledge (Cronbach’s α = 0.770; KMO = 0.759***)			Standard loading
Statement	Frequency	Percentage (%)	
COVID-19 is a viral disease.			0.938
No Yes Not sure	113 331 143	19.3 56.4 24.4	
Children and youngsters should not take actions to prevent the infection by the COVID-19.			0.891
No Yes Not sure	350 168 69	59.6 28.6 11.8	
To prevent COVID-19, everyone should avoid densely packed places, for example train stations and public transportation.			0.962
No Yes Not sure	0 551 36	0 93.9 6.1	
Successful measures of decreasing the spread of COVID-19 are via isolation and care of individuals who are contaminated by COVID-19.			0.861
No Yes Not sure	124 337 86	21.1 64.2 14.7	
Individuals who contact persons infected with COVID-19 should isolate separately. Generally, the isolation time is 14 days.			0.900
No Yes Not sure	24 460 103	4.1 78.4 17.5	
Common cold, stuffy nose, runny nose, and sneezing are uncommon in individuals infected by COVID-19.			0.845
No Yes Not sure	220 22 345	37.5 3.7 58.8	
Currently there is no successful cure for COVID-19, but timely advice and helpful action can assist most victims to get better from the disease.			0.927
No Yes Not sure	87 466 34	14.8 79.4 5.8	
The older adult and persons with underlying chronic diseases are at risk of rigorous infection and death.			0.803
No Yes Not sure	146 306 35	24.9 52.1 23	
COVID-19 can be transferred by eating or touching wild animals.			0.748
No Yes Not sure	121 268 198	20.6 45.7 33.7	
Individuals who are infected by COVID-19 cannot contaminate others while fever is not there.			0.874
No Yes Not sure	467 79 41	79.6 13.5 7	
Hygienic standard hand wash and using facemasks are primary prevention methods of COVID-19 spread.			0.977
No Yes Not sure	36 526 25	6.1 89.6 4.3	
COVID-19 can be transferred to individuals who touch other individuals infected by the virus and then touch their own face.			0.928
No Yes Not sure	0 562 25	0 95.7 4.3	
An individual can be infected by COVID-19 via insect bite.			0.777
No Yes Not sure	74 211 302	12.6 35.9 51.4	
An individual can be infected by COVID-19 via water and meals.			0.666
No Yes Not sure	294 163 130	50.1 27.8 22.1	
An individual can be infected by COVID-19 though matters infected by coronavirus.			0.745
No Yes Not sure	185 312 90	31.5 53.2 15.3	
Hand cleaning, covering the nose and mouth while coughing, and keeping away from ill contacts can help with the avoidance of COVID-19 spread.			0.800
No Yes Not sure	108 479 0	18.4 81.6 0	
COVID-19 can be transferred from creature to individual.			0.891
No Yes Not sure	134 314 139	22.8 53.5 23.7	
COVID-19 cause pneumonia and respiratory difficulty and leads to death.			0.959
No Yes Not sure	95 378 114	16.2 64.4 19.4	
COVID-19 is spread via air and making contact with fecal-oral routes.			0.754
No Yes Not sure	75 285 227	12.8 48.6 38.7	
COVID-19 can be transferred from individual to individual via a short distance.			0.878
No Yes Not sure	92 462 33	15.7 78.7 5.6	
COVID-19 can be spread by contact with a surface contaminated by the virus and touching the mouth, nose, and eyes.			0.835
No Yes Not sure	0 511 76	0 87.1 12.9	
All individuals with COVID-19 do not have dangerous cases. Only elderly adults with lifelong sickness tend to have harsh cases.			0.834
No Yes Not sure	271 112 204	46.2 19.1 34.8	
Pregnant women are more at risk of contamination than non-pregnant women.			0.965
No Yes Not sure	314 126 147	53.5 21.5 25	
Children are not at greater risk for COVID-19 than adults.			
No Yes Not sure	302 119 166	51.4 20.3 28.3	0.898

***Significant at *p* < 0.001.

### Respondents’ attitude toward COVID-19

The average attitude score for COVID-19 was 63.2 (S.D. = 4.6, min = 19, max = 76). Cronbach’s α for the attitude scale was 0.802, indicating that the questionnaires were reliable. Furthermore, the KMO values were 0.753, which shows that the criteria of validity are met. All the items in attitude satisfied the standard loading value of >0.40. Overall, 65.9% of the study participants had scored an attitude score greater than the mean attitude score (63.2) and had a positive attitude toward COVID-19. In total, 77% of the study participants agreed to take a vaccine for COVID-19 when available, 79 (13.5%) respondents agreed to welcome friends and family with a handshake; 312 (53.2%) agreed that washing hands was the necessary action to prevent the infection, 340 (57.9%) agreed that sidestepping from the individuals was the best way to prevent the disease, 396 (67.5%) agreed to clear their hands frequently and for a sufficient period of time to prevent the infection, and 368 (62.7%) agreed to wear a facemask to prevent the infection (Table 4).

**Table 4:** Participants’ attitude on COVID-19 in Hawassa city, Sidama, Ethiopia, 2020 (*n* = 587).

Items related to attitude (Cronbach’s α = 0.802; KMO = 0.753***)			Standard loading
Statement	Frequency	Percentage (%)	
When I get together with friends and family, I will always welcome them with a handshake.			0.568
Strongly disagree Disagree Agree Strongly agree	251 256 79 1	42.8 43.6 13.5 0.2	
When I get together with friends and family, I will always welcome them with a hug.			0.569
Strongly disagree Disagree Agree Strongly agree	138 230 217 2	23.5 39.2 37 0.3	
I clean my hands frequently and for a sufficient period of time.			0.617
Strongly disagree Disagree Agree Strongly agree	5 50 396 136	0.9 8.5 67.5 23.2	
I regularly wear a facemask to defend myself from the danger of virus.			0.796
Strongly disagree Disagree Agree Strongly agree	0 19 368 200	0 3.2 62.7 34.1	
If I see an individual contaminated with the infection, I will tell the public health service.			0.722
Strongly disagree Disagree Agree Strongly agree	0 97 230 260	0 16.5 39.2 44.3	
If I feel any of the indications linked with the infection, I will tell the public health service.			0.803
Strongly disagree Disagree Agree Strongly agree	1 118 230 238	0.2 20.1 39.2 40.5	
If I touch an individual contaminated with the disease, I consent to be isolated at home for a definite period of time until it is confirmed that I am free from the sickness.			0.853
Strongly disagree Disagree Agree Strongly agree	0 136 209 242	0 23.2 35.6 41.2	
If I touch an individual contaminated with the disease, I consent to be isolated at quarantine facility for a certain period of time until it is confirmed that I am completely free from the sickness.			0.829
Strongly disagree Disagree Agree Strongly agree	0 107 274 206	0 18.2 46.7 35.1	
If I am requested to be isolated for some period of time, I believe that my wage should be carried on in this period.			0.677
Strongly disagree Disagree Agree Strongly agree	8 152 171 256	1.4 25.9 29.1 43.6	
If an immunization is obtainable for the infection, I am ready to get it.			0.752
Strongly disagree Disagree Agree Strongly agree	17 118 205 247	2.9 20.1 34.9 42.1	
I regularly track updates about the spread of the infection in my country.			0.64
Strongly disagree Disagree Agree Strongly agree	0 21 213 353	0 3.6 36.3 60.1	
I regularly track updates about the spread of the infection globally.			0.782
Strongly disagree Disagree Agree Strongly agree	0 29 306 252	0 4.9 52.1 42.9	
If a talk about the infection is prepared around me, I will be there.			0.720
Strongly disagree Disagree Agree Strongly agree	2 90 242 253	0.3 15.3 41.2 43.1	
If brochures are distributed that contains facts about COVID-19, I am ready to read them and follow the teachings introduced in them.			0.751
Strongly disagree Disagree Agree Strongly agree	0 63 327 197	0 10.7 55.7 33.6	
If defensive measures and tools are obtainable at a reasonable cost, I will purchase them.			0.739
Strongly disagree Disagree Agree Strongly agree	1 17 313 256	0.2 2.9 53.3 43.6	
It is necessary to sidestep from others to evade the spread of COVID-19.			0.710
Strongly disagree Disagree Agree Strongly agree	0 29 340 218	0 4.9 57.9 37.1	
Hand washing is necessary to defend myself from COVID-19.			0.894
Strongly disagree Disagree Agree Strongly agree	0 21 312 254	0 3.6 53.2 43.3	
To defend myself from COVID-19 contact, I should stay at home.			0.666
Strongly disagree Disagree Agree Strongly agree	0 98 316 173	0 16.7 53.8 29.5	
COVID-19 will finally be effectively managed.			0.635
Strongly disagree Disagree Agree Strongly agree	55 166 233 133	9.4 28.3 39.7 22.7	

***Significant at *p* < 0.001.

Variables that were identified as significant in the univariate analysis were included in the multivariate analysis and shown in Table 5. Educational level with categories second degree and above (AOR = 29.709, 95% CI = 1.239–12.55), first degree (AOR = 3.476, 95% CI = 3.278–22.02), certificate/diploma (AOR = 3.37, 95% CI = 1.062–18.24), and grade 12 completed (AOR = 1.903, 95% CI = 2.12–6.809) were the significant variables that were correlated with the knowledge of the respondents. Age of the respondents with categories above 50 (AOR = 1.545, 95% CI = 2.513–4.658), 40–50 (AOR = 1.542, 95% CI = 1.847–2.809), 29–39 (AOR = 0.849, 95% CI = 2.518–1.394) were the significant variables that correlated with attitude.

**Table 5:** Association of explanatory variables with knowledge and attitude of residents at Hawassa city at Sidama region, Ethiopia, 2020 (*n* = 587).

Variable	Categories	Knowledge AOR (CI: 95%)	Attitude AOR (CI: 95%)
Age in years	18–28	1	1
	28–38	0.993 (0.271–3.630)	0.849 (2.518–1.394)*
	38–48	1.035 (0.125–8.548)	1.542 (1.847–2.809)*
	Above 48	1.331 (0.038–2.895)	1.545 (2.513–4.658)*
Gender	Man	1	1
	Woman	0.927 (0.330–2.61)	0.774 (0.506–1.185)
Marital status	Single	1	1
	Married	2.501 (0.714–8.754)	2.690 (1.127–6.234)*
	Divorced		3.700 (0.844–2.745)*
Educational level	No formal education	1	1
	Up to and 12 com	1.903 (2.12–6.809)*	1.188 (0.707–1.997)
	Certificate/diploma	3.37 (1.062–18.24)*	2.769 (1.448–5.155)*
	Degree	3.476 (3.278–22.02)*	3.875 (0.232–6.275)*
	M.Sc. and above	29.709 (1.239–12.55)*	3.997 (1.008–5.274)
Employment status	Unemployed	1	1
	Employed	10.053 (1.783–56.673)*	0.607 (0.232–1.591)
	Self-employed	9.545 (1.165–78.173)*	0.981 (0.368–2.619)
Monthly income in birr	Under 1,000	1	1
	1,000–3,000	0.109 (0.225–5.460)	2.282 (1.009–5.163)*
	3,001–6,000	0.466 (0.142–4.134)	2.690 (1.127–6.423)*
	Above 6,000	1.763 (0.183–11.662)	3.811 (1.452–9.943)*
Accommodation	Living alone	1	1
	Not living alone	0.847 (0.128–5.602)	1.472 (0.822–2.635)
Total family size	1–4	1	1
	5–8	0.666 (0.081–5.498)	1.622 (1.879–2.993)*
	Above8	0.988 (0.139–7.009)	2.158 (1.128–4.129)*
Reading habit	Rarely	1	1
	Sometimes	17.24 (17.213–1661.966)*	2.512 (0.319–0.822)*
	Always	34.45 (26.608–4462.226)*	5.625 (0.057–0.833)*
Use of social media	Rarely	1	1
	Sometimes	3.432 (3.504–23.378)*	2.367 (0.235–0.573)*
	Always	38.708 (5.086–294.610)*	3.891 (0.144–5.681)

*Significant.

## Discussion

This research attempted to evaluate the level of knowledge and attitude of the general population of Hawassa city toward COVID-19 and the interaction among knowledge and attitude toward COVID-19 from 3 June 2020 to 30 August 2020.

The majority of the participants (61.7%) were knowledgeable about COVID-19. This result is much lower compared to other studies that have been done on Saudi Arabian residents (97%) who were knowledgeable regarding COVID-19 (11, 12). Another research that has been done in Iran indicated that 56.5% of the study participants were knowledgeable regarding COVID-19 (13). Our study result was much lower compared to other studies conducted in the Chinese general population (90%) and the United States (80%) (6, 14). This difference might be due to the difference in the study period or the difference in questions that measure the knowledge and attitude of the respondents.

Knowledge about the symptoms of COVID-19 was 62.5%. This result was much lower compared to other studies conducted in Iran’s general population (80%) (15). In this study, some misconceptions about the disease persisted. For example, 35.9% of the participants believe that the virus can be spread by insect bite and 53.5% believe that the virus is transmitted via animals. This study result was almost similar to the study done in Henan, China (16). In our setting, education and more detailed comprehensive training were vital in improving the knowledge of participants about COVID-19.

The majority of the study participants (65.9%) showed a positive attitude toward COVID-19. A probable reason is that good knowledge about COVID-19 among participants may be a guide to a positive attitude. This positive attitude was improved by a high association between the knowledge and attitude of the respondents (*chi-square* = 22.644, *p* = 0.00), which was statistically significant. This result is in line with the study (17) that found that more than 60% of respondents have a positive attitude toward COVID-19. In our study, gender, marital status, employment status, and accommodation had no significant association with the attitude of participants toward COVID-19. These results are in line with a study done in the United States (14), which indicated that the attitude regarding COVID-19 was not associated with marital status, gender, and employment status.

Based on logistic regression analysis, female respondents were more likely to be knowledgeable about COVID-19 than male respondents, but the difference was not significant. Pearson’s correlation coefficient revealed significant positive linear correlations between knowledge and attitude, which was significant. This correlation may be explained by reasoned action theory. The theory states that a person’s intention to undertake a specific behavior is a function of their attitude toward that behavior (18). In total, 79.4% of the participants knew that there was no successful cure for COVID-19 as of the date of this study. Viral contaminations have been recognized to be highly infectious among people in close contact (19). However, approximately 21.3% of the respondents were unaware that COVID-19 could transfer from individual to individual via a short distance. It was also evident that, of the current general population, 48.6% were unaware that children are not at less risk for COVID-19 than adults. According to the WHO, all persons are susceptible to COVID-19 (13). These findings highlight the need to continue to encourage and emphasize maintaining social distancing and creating knowledge. The study recommends that health ministries should provide an ample training program, targeting younger age groups, women, and lower income groups to promote all precautionary and defensive measures of COVID-19 to achieve balance in terms of knowledge about COVID-19.

Some of the significant factors that are associated with the knowledge of the participants were educational level, employment status, reading habits, and uses of social media. This finding is supported by other studies that identified employed persons, those with higher income levels, and more educated respondents who are more knowledgeable about emerging communicable diseases (11, 20). Approximately 62.4% of the study participants agreed that the virus can be successfully controlled. This finding is consistent with a recent study conducted in China, where the majority of participants were convinced that the disease is curable and that their country will combat the disease (6).

Women were more likely to have knowledge and optimistic attitudes toward COVID-19. These findings are consistent with other studies showing that, in response to SARS and MERS, men were significantly less likely to take preventive and protective measures than women (15). Almost 90.7, 96.8, and 86.4% of the participants believe that cleaning hands, regularly wearing a facemask, and avoiding shaking hands, respectively, are methods to control the spread of the infection. Respondents had good knowledge and a positive attitude as a result of Ethiopian health authorities providing education and outreach materials to boost public understanding of the infection. Finally, the study findings may be useful to inform policymakers and healthcare professionals on further public health interventions, knowledge raising, policies, and health education programs.

### Limitations

The data existing in this study are self-reported and somewhat dependent on the respondents’ honesty and recall ability; thus, they may be subject to recall unfairness. Future research might employ administrative data to address this issue. Regardless of these limitations, the study findings provide valuable information about the knowledge and attitudes of residents.

## Conclusion

Our results indicate that Hawassa city residents, especially men, older persons, the educated ones, readers of newspapers, and followers of mass media such as television and radio, have good knowledge and positive attitudes toward COVID-19. Although the general knowledge and attitude of respondents toward COVID-19 was positive, there is a need to use more effective strategies to improve knowledge and attitude toward COVID-19, and knowledge creation on preventive behaviors among the community is highly recommended to attain better results. The educational level, use of social media, and reading habits of the respondents appear to play significant roles in determining their level of knowledge and attitude toward COVID-19. The results of this study suggest that more emphasis should be placed on less educated persons, lower income persons, women, and younger persons. The results may assist policymakers in recognizing the target populations for COVID-19 prevention.

## Data availability statement

The original contributions presented in the study are included in the article/supplementary material, further inquiries can be directed to the corresponding author.

## Ethics statement

Ethical clearance (DRBH/125/2020) was obtained from the Department Review Board of Hawassa University. The reason and significance of the study were explained, and informed written consent was obtained from the respondents before conducting the study.

## Author contributions

BW: Conceptualization, Data curation, Formal analysis, Investigation, Methodology, Software, Supervision, Validation, Visualization, Writing – original draft.
